# Quality Assessment of Digital Health Applications: Protocol for a Scoping Review

**DOI:** 10.2196/36974

**Published:** 2022-07-20

**Authors:** Godwin Denk Giebel, Nils Frederik Schrader, Christian Speckemeier, Carina Abels, Kirstin Börchers, Jürgen Wasem, Nikola Blase, Silke Neusser

**Affiliations:** 1 Institute for Healthcare Management and Research University of Duisburg-Essen Essen Germany; 2 QM BÖRCHERS CONSULTING+ Herne Germany

**Keywords:** digital health application, mHealth, mHealth app, quality assurance, quality assessment, mobile health, application, protocol, app, digital health, telehealth, eHealth

## Abstract

**Background:**

All over the world, development and usage of mobile health (mHealth) apps is increasing. While apps offer numerous opportunities to improve health care, there are associated problems that differ significantly from those of traditional health care services. Further investigations on the quality of mHealth apps are needed to address these problems.

**Objective:**

This study aims to identify and map research on quality assessment and quality assurance of mHealth apps and their transferability to continuous quality assurance of mHealth apps.

**Methods:**

The scoping review will follow published methodological frameworks for scoping studies as well as Preferred Reporting Items for Systematic reviews and Meta-Analyses extension for Scoping Reviews criteria. Electronic databases (Medline, EMBASE, and PsycINFO), reference lists of relevant articles, and websites of relevant institutions will be searched. Two reviewers will independently assess eligibility of articles. Therefore, a 2-stage (title and abstract, followed by full text) screening process was conducted. Quality management systems and quality assessment tools will be analyzed and included in our review. Particular focus is placed on quality dimensions.

**Results:**

This scoping review provides an overview of the available evidence and identifies research gaps regarding continuous quality assessment of mHealth apps. Thereby, relevant quality dimensions and criteria can be identified and their eligibility and relevance for the development of a continuous quality assurance system of mHealth apps can be determined. Our results are planned to be submitted to an indexed, peer-reviewed journal in the second half of 2022.

**Conclusions:**

This is the first review in the context of continuous quality assurance of mHealth apps. Our results will be used within the research “Continuous quality assurance of Digital Health Applications” (“QuaSiApps”) project funded by the German Federal Joint Committee.

**International Registered Report Identifier (IRRID):**

DERR1-10.2196/36974

## Introduction

Since the introduction of the Apple app Store (iOS) in 2008, mobile health (mHealth) apps have become increasingly popular. Today, inter alia promoted by the COVID-19 pandemic in 2020, the implementation of mHealth apps is even accelerated [1]. Overall, more than 350,000 health and fitness or medical apps are available from Apple App Store (iOS) and Google Play (Android) [1]. Many of them are not extensively tested for quality, mainly because they do not fall under medical device regulation [2].

In 1966, Donabedian [3] suggested a framework to evaluate the quality of medical care, consisting of an examination of structure, process, and outcome quality. While the assignment of quality criteria to these dimensions is in most cases straightforward, the identification of valuable quality criteria is often difficult. According to the International Organization for Standardization (ISO) 9000 standard, quality is defined as “the degree to which a set of inherent characteristics fulfills requirements” [4]. Given this definition, the assessment of quality brings a 3-fold challenge. First, the set of relevant characteristics has to be defined. Second, instruments are needed to measure the relevant characteristics. Third, the levels of requirements have to be defined. Mastering this challenge and implementing a quality management system is a strategic decision by a health care organization, which can help improve its overall performance and provide a good foundation for sustainable development initiatives [5].

Nowadays, international quality standards exist in health care. Those are, for example, quality management systems in health care (Deutsches Institut fur Normung [DIN] Europaische Norm [EN] 15224:2016 in connection with ISO 9001:2015), processes to analyze the risk to the quality, and safety of health care and continuity of care when telehealth services are used to support health care activities [6] and quality management systems specific to the medical device industry [7]. Owing to the importance of quality management in the context of health care, especially in the context of telehealth services and the medical device industry, further investigations on the quality of mHealth apps are needed.

This scoping review is one module of a larger research project. The overall project “Continuous quality assurance of Digital Health Applications” (“QuaSiApps”) aims to develop a continuous quality assurance system for approved and refundable mHealth apps (“DiGA”) in the German health care system [8]. The research project is funded by the German Federal Joint Committee (G-BA) [9].

The aim of this scoping review is to map the research conducted in the field of continuous quality assessment and quality assurance of mHealth apps. This includes quality management systems, quality dimensions, rating scales, quality measurement tools, quality criteria, as well as quality requirements to assess the quality of mHealth apps and their transferability to continuous quality assurance systems.

## Methods

### Overview

The review process will follow the 5 stages described by Arksey and O’Malley [10] and enhanced by Levac et al [11]: (1) identifying the research question (completed), (2) identifying relevant studies (completed), (3) selecting studies (ongoing), (4) charting the data and collating, and (5) summarizing and reporting the results. The manuscript will be prepared in accordance with the PRISMA-ScR (Preferred Reporting Items for Systematic reviews and Meta-Analyses extension for Scoping Reviews) [12].

### Data Sources and Search Strategy

The search strategy was developed in accordance with the JBI Manual for Evidence Synthesis concept describing 3 steps [13]. First, an initial limited search was conducted in EMBASE and Medline to search for relevant search terms contained in the title, abstract, or key words. Second, identified key words were combined and used as search queries in EMBASE, Medline, and additionally in PsycINFO. Finally, reference lists of articles included after full-text screening were screened for eligibility of inclusion.

On July 26, 2021, EMBASE, Medline, and PsycINFO were searched with the following systematic search string: ((((“assessment*” OR “*evaluation*” OR “*measurement*” OR “*score*” OR “*criteri*a” OR “*scale*”) AND “*quality*”) OR (“*quality assurance*” OR “*quality indicators*” OR “*quality control*” OR “*quality assessment tool*” OR “*health care quality*” OR “*quality improvement*”) OR (“*norm*” OR “framework” OR “*guideline*”)) AND (“*web application*” OR “*mobile application*” OR “*mHealth*” OR “*virtual care*” OR “*healthcare app*” OR “*health care app*” OR “*mobile health*” OR “*health app*” OR “*smartphone application*”) AND (“*healthcare*” OR “*health care*”)).

The individual search terms were restricted to abstract, title, and key word search but expanded by indexing terms (MeSH and Emtree) as well as truncations. The appendix of this protocol includes the precise search strategy (Multimedia Appendices 1-3).

Articles were not included if the language was not English or German, and the search was limited to articles published between January 1, 2016, and July 8, 2021. A justification and explanation of the restrictions is given in the discussion of this protocol.

Besides the systematic search in databases, a structured study will be performed to discover gray literature, guidelines, and working papers from various governmental and nongovernmental institutions (Multimedia Appendix 4). Furthermore, relevant ISO and DIN standards will be included.

### Inclusion and Exclusion Criteria

An explorative search, 2 publications [14,15], and internal discussion helped us develop inclusion and exclusion criteria (Textbox 1).

**Inclusion and exclusion criteria.**

**Inclusion criteria**

- Articles including the following:
  development or
  description or
  application and description or
  validation and description or
  review (systematic) or
  intended use, institutional linkage, or type of reporting of disease independent concepts of quality assessment or quality assurance in mobile health (mHealth) apps
- The investigated mHealth apps must fulfill the following criteria:
  used by the patient and
  used in outpatient treatment and
  with more functions than the following: improvement of adherence, text-messaging, reminder or screening for primary prevention or (video) consultation or Disease education or Reading out and controlling of devices
- Language: English and German
- Articles published in 2016 or afterwards

**Exclusion criteria**

- The investigated mHealth app fulfills one of the following criteria:
  health care practitioner use or
  inpatient treatment or
  with not more functions than the following: improvement of adherence, text-messaging, reminder or screening for primary prevention or (video) consultation or Disease education or Reading out and controlling of devices
- Research protocols, conference abstracts, letters to the editor, or expression of opinions

### Study Screening and Selection

Identified citations were imported in Endnote X9 (Clarivate Analytics). After removing duplicates, our search strategy resulted in 2235 articles for title and abstract screening. Two reviewers (GG and NS) independently assessed the titles and abstracts of these articles to decide whether an article is eligible for full-text screening or not. Full-text screening and assessment against inclusion and exclusion criteria was conducted by the same reviewers. Reasons for noninclusion and exclusion were captured.

Articles assessed as eligible for the purpose of our review will be included and relevant information and (meta-)data will be extracted and summarized. Multimedia Appendix 5 provides a preliminary outlook on extracted information categories (Multimedia Appendix 5). Procedural purposes and usage of quality management systems are extracted separately.

In case of disagreement between the 2 reviewers, a third person (SN) will join the discussion and decide whether a text is eligible or not for inclusion. In case of missing data or uncertainty, the reviewers will contact authors of included papers.

## Results

A structured search strategy was developed to find and summarize evidence for continuous quality measuring and quality assurance in the context of mHealth apps. Results of this search will be presented in the form of a scoping review. Flowcharts will be used to depict the process of article selection, and extracted data of included articles will be presented in tables as well as a narrative summary.

## Discussion

This scoping review will identify concepts and studies of quality assessment and quality assurance of mHealth apps. Once a concept or study is identified and included, relevant quality dimensions and criteria will be extracted. The evidence thus gathered will be systematized by categorizing the extracted dimensions and criteria in overarching quality dimensions. Based on this, we will assess the relevance and transferability of extracted dimensions for continuous quality assurance.

Thereby, along with Arksey and O'Malley [10], we will summarize the evidence on continuous quality assurance of mHealth apps. This will also provide an overview on research gaps in the literature [10]. As part of our QuaSiApps project, the results will be used alongside those of another scoping reviews [16], focus groups, and stakeholder surveys to develop a continuous quality assurance system.

Regarding prior work, there are some aspects, such as usability or data privacy, that are well studied in the field of quality assurance of mHealth apps in general. However, which of these or other quality dimensions have relevance for continuous quality assurance remains unanswered. Furthermore, quality assessment in health care is based on the measurement of quality criteria [17]. However, methods of quality assessment of mHealth apps are heterogeneous. Azad-Khaneghah et al [15] reviewed mHealth usability and quality rating scales and compared them in terms of purpose, content, and intended target users. Nouri et al [14] additionally extracted and classified the criteria for assessing the quality of mHealth apps out of assessment tools or methods. Our review aims to incorporate the most recent evidence in this fast-moving environment and especially aims to shed light on concepts, guidelines, and working papers published by governmental and nongovernmental institutions. Further, our review is part of a larger research project focusing on continuous quality assurance in the German mHealth context. Thus, an emphasis will be placed on the determination of aspects involving continuous quality assurance.

However, besides usability and data privacy, quality assessment and continuous quality assurance in mHealth apps remains largely unexamined. Especially in the rapid evolving and changing field of mHealth apps, the development of a continuous quality assurance system is essential to guarantee high quality even beyond the app development or app approval to guarantee a safe and sustainable use.

Our scoping review has 2 major limitations owing to resource limitations. First, it includes only articles published after 2016. Second, articles were only considered if their language was English or German. The restriction of time is justified by the fact that Nouri et al [14] cover earlier relevant evidence about criteria for assessing the quality of mHealth apps in their systematic review. If our review does not cover all criteria reported by Nouri et al [14], we shall supplement our review with their findings. The language restriction was made because English and German were the only common languages of the reviewers. Regarding the search strategy of the proposed scoping review, it should be considered that there is still inconsistency regarding terminology [16].

Therefore, we pilot-tested different terms and compared the results to guarantee a valid search strategy. While we use the term “Application” in accordance with the German Federal Institute for Drugs and Medical Devices, a consensus paper recommends the use of “App” [18].

This scoping review gathers existing studies and concepts on quality assessment and quality assurance of mHealth apps. This will be a basis for the development of a continuous quality assurance system for mHealth apps. It helps to identify the relevant quality dimensions, which should be considered in such a concept.

### Conclusions

This scoping review will provide deeper insight into the field of quality measurement and quality assurance in the context of mHealth apps. It will provide an overview of relevant quality dimensions and quality criteria especially with relevance for continuity. Our research findings can serve as a fundament for the development of continuous quality assurance systems.

## Appendix

Multimedia Appendix 1 Search strategy; Embase.

Multimedia Appendix 2 Search strategy Medline via Ovid.

Multimedia Appendix 3 Search strategy PsycINFO via Ovid.

Multimedia Appendix 4 Sources of gray literature.

Multimedia Appendix 5 Data extraction.

## Abbreviations

DIN: Deutsches Institut fur Normung
EN: Europaische Norm
G-BA: German Federal Joint Committee
mHealth: mobile health
PRISMA-ScR: Preferred Reporting Items for Systematic reviews and Meta-Analyses extension for Scoping Reviews
QuaSiApps: Continuous quality assurance of Digital Health Applications
