# Supplementary material for: Quality Assessment of Digital Health Applications: Protocol for a Scoping Review
Source: JMIR Res Protoc. 2022 Jul 20;11(7):e36974. doi: 10.2196/36974 (PMC9350825; doi:10.2196/36974)
Supplement: Multimedia Appendix 1 [file resprot_v11i7e36974_app1.docx]

## Search strategy; Embase

| Database | EMBASE | |
| --- | --- | --- |
| Platform | **Embase.com** | |
| Date of search | **26.07.2021** | |
| Filter | **No filters** | |
| # | Search Term | Hits |
| 1a | asses*:ab,ti,kw | 4,633,449 |
| 1b | evaluat*:ab,ti,kw | 5,364,243 |
| 1c | measur*:ab,ti,kw | 4,811,688 |
| 1d | score*:ab,ti,kw | 1,620,727 |
| 1e | scoring:ab,ti,kw | 139,094 |
| 1f | criteri*:ab,ti,kw | 1,120,714 |
| 1g | scale*:ab,ti,kw | 1,213,789 |
| 1h | scaling*:ab,ti,kw | 54,382 |
| 1i | **(1a + 1b + 1c + 1d + 1e + 1f + 1g + 1h + 1i + 1j + 1k + 1l + 1m +1n)** | 12,429,766 |
|  |  |  |
| 2a.1 | quality:ab,ti,kw | 1,598,945 |
| 2a.2 | quality/exp/mj | 2 |
| 2a | **quality:ab,ti,kw OR quality/exp/mj** | 1,598,945 |
| 2b | **1i AND 2a** | 1,064,331 |
|  |  |  |
| 3a | 'quality assurance':ab,ti,kw | 42,357 |
| 3b | 'quality indicators':ab,ti,kw | 11,938 |
| 3c | 'quality control':ab,ti,kw | 77,476 |
| 3d | 'quality assessment tool':ab,ti,kw | 1,457 |
| 3e | 'health care quality':ab,ti,kw | 4,401 |
| 3f | 'quality improvement':ab,ti,kw | 67,426 |
|  |  |  |
| 3h | 'quality control'/exp/mj | 97,568 |
| 3i | 'quality assessment tool'/exp/mj | 246 |
| 3j | 'health care quality'/exp/mj | 614,014 |
| 3k | 'quality improvement'/exp/mj | 24,646 |
|  |  |  |
| 3f | **(3a + 3b + 3c + 3d + 3e + 3f + 3g + 3h + 3i + 3j + 3k)** | 67,135 |
|  |  |  |
| 4a | **(2b + 3f)** | 1,102,149 |
|  |  |  |
| 5a | norm:ab,ti,kw | 25,576 |
| 5b | framework:ab,ti,kw | 319,322 |
| 5c | guideline:ab,ti,kw | 105,001 |
|  |  |  |
| 5d | **(5a + 5b + 5c)** | 445,629 |
|  |  |  |
| 6a | **(4a + 5d)** | 1,500,342 |
|  |  |  |
| 7a | 'web application':ab,ti,kw | 2,997 |
| 7b | 'mobile application':ab,ti,kw | 3,292 |
| 7c | mHealth:ab,ti,kw | 6,150 |
| 7d | 'virtual care':ab,ti,kw | 504 |
| 7e | 'healthcare app':ab,ti,kw | 14 |
| 7f | 'health care app':ab,ti,kw | 13 |
| 7g | 'mobile health':ab,ti,kw | 5,808 |
| 7h | 'health app':ab,ti,kw | 402 |
| 7i | 'smartphone application':ab,ti,kw | 2,665 |
|  |  | 8,385 |
| 7j | 'mobile application'/exp/mj |  |
|  |  |  |
| 7j | **(7a + 7b + 7c + 7d + 7e + 7f + 7g + 7h + 7i + 7j)** | 22,328 |
|  |  |  |
| 8a | healthcare:ab,ti,kw | 406,602 |
| 8b | 'health care':ab,ti,kw | 488,305 |
| 8c | **(11a + 11b)** | 853,548 |
|  |  |  |
| 9a | **[2016-2021]/py** | 9,122,486 |
|  |  |  |
| 10a | **6a AND 7j AND 8c AND 9a** | 1,084 |

((((asses*:ab,ti,kw OR evaluat*:ab,ti,kw OR measur*:ab,ti,kw OR score*:ab,ti,kw OR scoring:ab,ti,kw OR criteri*:ab,ti,kw OR scale*:ab,ti,kw OR scaling*:ab,ti,kw)

AND

(quality:ab,ti,kw OR quality/exp/mj))

OR

('quality assurance':ab,ti,kw OR 'quality indicators':ab,ti,kw OR 'quality control':ab,ti,kw OR 'quality assessment tool':ab,ti,kw OR 'health care quality':ab,ti,kw OR 'quality improvement':ab,ti,kw OR 'quality control'/exp/mj OR 'quality assessment tool'/exp/mj 'health care quality'/exp/mj OR 'quality improvement'/exp/mj))

OR

(norm:ab,ti,kw OR 'framework':ab,ti,kw OR guideline:ab,ti,kw))

AND

('web application':ab,ti,kw OR 'mobile application':ab,ti,kw OR mHealth:ab,ti,kw OR 'virtual care':ab,ti,kw OR 'healthcare app':ab,ti,kw OR 'health care app':ab,ti,kw OR 'mobile health‘:ab,ti,kw OR 'health app':ab,ti,kw OR 'smartphone application':ab,ti,kw OR 'mobile application'/exp/mj)

AND

(healthcare:ab,ti,kw OR 'health care':ab,ti,kw)

AND

[2016-2021]/py
