# Supplementary material for: Quality Assessment of Digital Health Applications: Protocol for a Scoping Review
Source: JMIR Res Protoc. 2022 Jul 20;11(7):e36974. doi: 10.2196/36974 (PMC9350825; doi:10.2196/36974)
Supplement: Multimedia Appendix 3 [file resprot_v11i7e36974_app3.docx]

## Search strategy PsycINFO via Ovid

| Database | PsycInfo | |
| --- | --- | --- |
| Platform | **Ovid** | |
| Date of search | **26.07.2021** | |
| Filter | **No filters** | |
| # | Search Term | Hits |
| 1a | asses*.mp | 878,652 |
| 1b | evaluat*.mp | 616,106 |
| 1c | measur*.mp | 944,098 |
| 1d | score*.mp | 362,136 |
| 1e | scoring.mp | 26,722 |
| 1f | criteri*.mp | 181,076 |
| 1g | scale*.mp | 729,826 |
| 1h | scaling*.mp | 14,582 |
| 1i | **(1a + 1b + 1c + 1d + 1e + 1f + 1g + 1h)** | 2,175,020 |
|  |  |  |
| 2a | quality.mp | 280,114 |
| 2b | **1i AND 2a** | 181,826 |
|  |  |  |
| 3a | "quality assurance".mp | 2,643 |
| 3b | "quality indicators".mp | 1,486 |
| 3c | "quality control".mp | 3,918 |
| 3d | "quality assessment tool".mp | 269 |
| 3e | "health care quality".mp | 846 |
| 3f | "quality improvement".mp | 6,441 |
|  |  |  |
| 3i | exp "quality control"/ | 1,811 |
|  |  |  |
| 3j | **(3a + 3b + 3c + 3d + 3e + 3f + 3g + 3h + 3i)** | 14,263 |
|  |  |  |
| 4a | **(2b + 3j)** | 186,744 |
|  |  |  |
| 5a | norm.mp | 16,720 |
| 5b | framework.mp | 192,479 |
| 5c | guideline.mp | 6,858 |
|  |  |  |
| 5d | **(5a + 5b + 5c)** | 214,474 |
|  |  |  |
| 6a | **(4a + 5d)** | 389,946 |
|  |  |  |
| 7a | "web application".mp | 201 |
| 7b | "mobile application".mp | 676 |
| 7c | mHealth.mp | 1,231 |
| 7d | "virtual care".mp | 42 |
| 7e | "healthcare app".mp | 0 |
| 7f | "health care app".mp | 0 |
| 7g | "mobile health".mp | 1,638 |
| 7h | "health app".mp | 86 |
| 7i | "smartphone application".mp | 455 |
|  |  |  |
| 7j | exp "mobile application"/ | 1,278 |
| 7k | exp "mHealth"/ | 1,255 |
|  |  |  |
| 7k | **(7a + 7b + 7c + 7d + 7e + 7f + 7g + 7h + 7i +7j + 7k)** | 3,857 |
|  |  |  |
| 8a | healthcare.mp | 50,718 |
| 8b | "health care".mp | 191,617 |
| 8c | **(11a + 11b)** | 221,885 |
|  |  |  |
| 9a | ("2016".yr OR "2017".yr OR "2018".yr OR "2019".yr OR "2020".yr OR "2021".yr) | 962,105 |
|  |  |  |
| 10a | **6a AND 7j AND 8c AND 9a** | 189 |

((((asses*.mp OR evaluat*.mp OR measur*.mp OR score*.mp OR scoring.mp OR criteri*.mp OR scoring.mp OR criteri*.mp OR scale*.mp OR scaling*.mp)

AND

(quality.mp))

OR

("quality assurance".mp OR "quality indicators".mp OR "quality control".mp OR "quality assessment tool".mp OR "health care quality".mp OR "quality improvement".mp OR exp "quality control"/))

OR

(norm.mp OR framework.mp OR guideline.mp))

AND

("web application".mp OR "mobile application".mp OR mHealth.mp OR "virtual care".mp OR "healthcare app".mp OR "health care app".mp OR "mobile health".mp OR "health app".mp OR "smartphone application".mp OR exp "mobile application"/ OR exp "mHealth"/)

AND

(healthcare.mp OR "health care".mp)

AND

("2016".yr OR "2017".yr OR "2018".yr OR "2019".yr OR "2020".yr OR "2021".yr)
