# Supplementary material for: Quality Assessment of Digital Health Applications: Protocol for a Scoping Review
Source: JMIR Res Protoc. 2022 Jul 20;11(7):e36974. doi: 10.2196/36974 (PMC9350825; doi:10.2196/36974)
Supplement: Multimedia Appendix 4 [file resprot_v11i7e36974_app4.docx]

## Sources of gray literature

| Country | Institution | Website |
| --- | --- | --- |
| Germany | Federal Ministry of Health / Bundesministerium für Gesundheit (BMG) | https://www.bundesgesundheitsministerium.de/ |
|  | Federal Joint Committee / Gemeinsamer Bundesausschuss (G-BA) | https://www.g-ba.de/ |
|  | Federal Institute for Drugs and Medical Devices / Bundesinstitut für Arzneimittel und Medizinprodukte (BfArM) | https://www.bfarm.de/ |
|  | Institute for Quality Assurance and Transparency in Healthcare / Institut für Qualitätssicherung und Transparenz im Gesundheitswesen (IQTIG) | https://iqtig.org/ |
| USA | U.S. Department of Health & Human Services (HHS) | https://www.hhs.gov/ |
|  | Agency for Healthcare Research and Quality (AHRQ) | https://www.ahrq.gov/ |
|  | Food and Drug Administration (FDA) | https://www.fda.gov/ |
| France | Ministère des solidarités et de la santé | https://solidarites-sante.gouv.fr/ |
|  | Haute autorité de santé | https://www.has-sante.fr/ |
| UK | Department of Health & Social Care | https://www.gov.uk/government/organisations/department-of-health-and-social-care |
|  | NHS | https://www.nhs.uk/ |
|  | National Institute for Health and Care Excellence (NICE) | https://www.nice.org.uk/ |
| Switzerland | Federal Office of Public Health / Bundesamt für Gesundheit (BAG) | https://www.bag.admin.ch/bag/en/home.html |
|  | Swiss Agency for Therapeutic Products / Schweizerisches Heilmittelinstitut (swissmedic) | https://www.swissmedic.ch/swissmedic/de/home.html |
|  | Swiss Competence and Coordination Centre of the Confederation and the Cantons (ehealthsuisse) | https://www.e-health-suisse.ch/startseite.html |
| International | World Health Organization (WHO) | https://www.who.int/ |
|  | Organisation for Economic Co-operation and Development (OECD) | https://www.oecd.org/ |
|  | European Medicines Agency (EMA) | https://www.ema.europa.eu/en |
