# Supplementary material for: Quality Assessment of Digital Health Applications: Protocol for a Scoping Review
Source: JMIR Res Protoc. 2022 Jul 20;11(7):e36974. doi: 10.2196/36974 (PMC9350825; doi:10.2196/36974)
Supplement: Multimedia Appendix 5 [file resprot_v11i7e36974_app5.docx]

Multimedia Appendix 5. Data extraction.

| Author | Year | Name of the instrument, tool, system, or concept | Short description | Quality dimensions |
| --- | --- | --- | --- | --- |
|  |  |  |  |  |
